# A predictive model for the early identification of patients at risk for a prolonged intensive care unit length of stay

**DOI:** 10.1186/1472-6947-10-27

**Published:** 2010-05-13

**Authors:** Andrew A Kramer, Jack E Zimmerman

**Affiliations:** 1 Senior Biostatistician, Cerner Corporation, 1953 Gallows Road, Suite 500, Vienna, Virginia 22182, USA; 2 Department of Anaesthesia and Critical Care Medicine, George Washington University, Washington, DC USA

## Abstract

**Background:**

Patients with a prolonged intensive care unit (ICU) length of stay account for a disproportionate amount of resource use. Early identification of patients at risk for a prolonged length of stay can lead to quality enhancements that reduce ICU stay. This study developed and validated a model that identifies patients at risk for a prolonged ICU stay.

**Methods:**

We performed a retrospective cohort study of 343,555 admissions to 83 ICUs in 31 U.S. hospitals from 2002-2007. We examined the distribution of ICU length of stay to identify a threshold where clinicians might be concerned about a prolonged stay; this resulted in choosing a 5-day cut-point. From patients remaining in the ICU on day 5 we developed a multivariable regression model that predicted remaining ICU stay. Predictor variables included information gathered at admission, day 1, and ICU day 5. Data from 12,640 admissions during 2002-2005 were used to develop the model, and the remaining 12,904 admissions to internally validate the model. Finally, we used data on 11,903 admissions during 2006-2007 to externally validate the model.

**Results:**

The variables that had the greatest impact on remaining ICU length of stay were those measured on day 5, not at admission or during day 1. Mechanical ventilation, PaO_2_: FiO_2 _ratio, other physiologic components, and sedation on day 5 accounted for 81.6% of the variation in predicted remaining ICU stay. In the external validation set observed ICU stay was 11.99 days and predicted total ICU stay (5 days + day 5 predicted remaining stay) was 11.62 days, a difference of 8.7 hours. For the same patients, the difference between mean observed and mean predicted ICU stay using the APACHE day 1 model was 149.3 hours. The new model's r^2 ^was 20.2% across individuals and 44.3% across units.

**Conclusions:**

A model that uses patient data from ICU days 1 and 5 accurately predicts a prolonged ICU stay. These predictions are more accurate than those based on ICU day 1 data alone. The model can be used to benchmark ICU performance and to alert physicians to explore care alternatives aimed at reducing ICU stay.

## Background

Length of stay is frequently used as a measure of ICU resource use, but there is no uniform definition of what constitutes a prolonged ICU stay [1,2]. A simplistic method uses the mean along with the standard deviation of length of stay for a population to assign a boundary of two standard deviations above the mean. But this is unsatisfactory, because the distribution of ICU length of stay is left-censored at zero and is heavily skewed to the right by a "tail" that represents patients with longer stays [3]. A prolonged ICU stay has also been subjectively defined by designating a specific duration of stay e.g., ≥ 10 days, ≥ 14 days, ≥ 21days, ≥ 30 days [4-7]. Another method for defining a prolonged stay is to visually examine the distribution of ICU stay in a population and identify a threshold for the "tail" that represents long stay patients [3,4].

Despite differences in definition, studies have repeatedly shown that a small percentage (7% to 11%) of lengthy ICU admissions account for a large proportion (40% to 50%) of resource use [8-10]. Because patients with a prolonged ICU stay consume a disproportionate amount of resources, their early identification can assist in improving unit efficiency. This is because identifying these individuals early can improve patient throughput by signalling a need for discharge planning or exploration of care alternatives. These alternatives might include palliative care consultation [11], early mobility therapy [12,13], transfer to an in-hospital chronic ventilator unit [14], or discharge to a long-term acute care facility [15,16].

We have previously described models for predicting overall ICU length of stay using day 1 patient data [10,17,18]. Although these models provide accurate benchmarks for assessing the efficiency of ICU throughput, the accuracy of prediction is reduced for patient groups with a prolonged ICU stay. This reduced accuracy has been attributed to uncertainty about prognosis, subsequent complications, and variations in response to therapy [10]. The same factors account, at least in part, for the reduced accuracy of hospital mortality prediction using ICU day 1 data [19,20].

Because models that include daily physiologic measures during therapy have improved the accuracy of daily mortality predictions [21-24], we examined whether a similar approach might improve predictions of ICU stay, particularly when length of stay is prolonged. Therefore, this study had the following objectives: 1) To identify a simple yet relevant clinical threshold for concern about a prolonged ICU stay. 2) To compare the characteristics of patients who stay less than or longer than the threshold for concern. 3) To describe the development and validation of a model for predicting ICU stay remaining after the threshold. 4) To compare the accuracy of ICU length of stay predictions on day 1 vs. a model that includes data collected on the threshold day.

## Methods

### Patient Cohort

Study data were retrospectively collected for consecutive admissions to U.S. intensive and coronary care units from January 1, 2002 to December 31, 2007. The hospitals and ICUs in this study were selected because each had installed an Acute Physiology and Chronic Health Evaluation (APACHE) computerized data collection and analysis system that recorded patient data on ICU days 1 to 7. Details about the characteristics of these hospitals, ICUs, and patients have been described elsewhere [20,25].

### ICU Length of Stay

ICU stay was measured using the exact interval (in minutes) between the day and time of ICU admission and ICU discharge, and converted back to fractional days (e.g. 3.12 days, 6.45 days). Exact ICU stay was used because previous studies have demonstrated superior accuracy compared to measurement using calendar days [10,26,27].

A frequency histogram of ICU length of stay was generated and a day selected to represent a clinical threshold when clinicians might be concerned that a patient will have a prolonged ICU stay. The threshold was chosen because: 1) it represented enough time to reflect complications and response to therapy for the patient; and 2) the proportion of patients still in the ICU at this point was approximately equal to the upper quintile for length of stay. Once the threshold day was selected, we developed and validated a model for predicting ICU stay remaining after the threshold day.

### Patient Data Collection

All patient data were generated as a result of patient care and entered on site using a software program that included computerized pick lists, automated error checking, and calculation of physiological means. Demographic and physiological data were entered via electronic interfaces with laboratory and clinical information systems. Data collected for each patient are shown in Table 1. Detailed descriptions of these demographic, clinical and physiological items have been previously reported [10,20,28].

**Table 1:** Information collected on all patients who remained in the intensive care unit (ICU) on day 5.

Variable	Measurement	Reference Group* (if applicable)
**Day 1**		

Acute Physiology Score (APS) Variables	Weight determined by most abnormal value within initial first APACHE day, sum of weights equals the APS, which ranges 0 to 252. Five spline terms added. Variables include pulse rate, mean blood pressure, temperature, respiratory rate, PaO_2 _: FiO2 ratio (or A-aD0_2 _for intubated patients with FIO2 > 0.5), hematocrit, white blood cell count, creatinine, urine output, blood urea nitrogen, sodium, albumin, bilirubin, glucose, acid base abnormalities, and neurological abnormalities based on Glasgow Coma Score. Continuous measure. Three spline terms added in the complex regression model.	N/A

Chronic Health Items (CHIs)	AIDS, cirrhosis, hepatic failure, immunosupression, lymphoma, leukemia or myeloma, metastatic tumor. Not used for elective surgery patients. Binary variable for > 0 CHIs. Complex model had a binary variable for each CHI separately.	N/A

ICU Admission Diagnosis	59 categories	Acute Myocardial Infarction

ICU Admission Source	Floor, operating/recovery room, other hospital, other admission source.	Direct admission

Length of Stay before ICU Admission	Square root. Two spline terms added in the complex regression model.	N/A

Age	Continuous measure. Three spline terms added in the complex regression model	N/A

PaO_2 _: F_i_O_2 _Ratio on Day 1	Divided by 10; Continuous measure. Three spline terms added in the complex regression model..	N/A

Emergency Surgery?	Binary	No

Ventilated on Day 1?	Binary	No

Unable to Assess Glasgow Coma Score on Day 1?	Binary	No

Thrombolytic Therapy (for patients with AMI)?	Binary	No

Rescaled Glasgow Coma Score on Day 1	12 - Glasgow Coma Score	N/A

Day 1 ICU Length of Stay Prediction	See reference (10)	N/A

Readmission to ICU?	Binary	No

**Day 5**		

Acute Physiology Score (APS) Variables	Same as for day 1 but taken on day 5. Continuous measure. Three spline terms added in the complex regression model.	N/A

Ventilated on Day 5?	Binary	No

Unable to Assess Glasgow Coma Score on Day 5?	Binary	No

PaO_2 _: F_i_O_2 _Ratio on Day 5	Divided by 10; Continuous measure	N/A

Rescaled Glasgow Coma Score on Day 5	12 - Glasgow Coma Score	N/A

Delta APS	APS on Day 4 - APS on Day 5	N/A

*"Reference Group" refers to the level of a discrete variable that was the default group used in the multivariable regression model.

Data collection procedures were based on prior reliability studies [29] and field experience [30,31], and details about data entry have been described elsewhere [10,20]. Based on contractual agreements between Cerner Corporation and each hospital, data were stripped of patient identifiers in full compliance with the Health Insurance Portability and Accountability Act (HIPAA) requirements. Informed consent was not obtained because these processes were identical to those that resulted in prior Institutional Review Board waivers [28].

We excluded patients admitted to ICUs that did not collect data during days 1 through 7. We also excluded patients who had been admitted for < 4 hours, patients with burns, and patients < 16 years of age. Patients admitted from or discharged to another ICU were excluded because of inability to determine their total ICU stays. We also excluded patients admitted after coronary artery bypass surgery because their ICU stay was generally short with a small range compared to other patients.

### Model Development and Validation

We developed a multivariable linear regression model to predict ICU stay remaining after the threshold day. A backwards elimination approach was used with a marginal p < 0.05 necessary to remain in the model. The model was intended for use in ICUs with advanced health information technology and the capability for automated data collection. The predictor variables in the regression model were collected on ICU day 1 and on the threshold day, and are displayed in Table 1. In addition, we used the change in Acute Physiology Score (APS) from the day before to the day of the threshold value. These variables were pre-selected based on previous research [10,20,22].

We extended the age, APS, and prior length of stay variables by including restricted cubic spline terms. Splines allow estimation of a non-linear relationship between a variable and ICU length of stay and replace less accurate techniques that assume a linear relationship [32,33].

The length of ICU stay was truncated at 30 days for patients with an ICU stay >30 days. We did this because a few outliers with an extremely long ICU stay can markedly distort a length of stay analysis [4]. The use of truncation at 30 days is also supported by prior studies [10,17,18,27,34], and by visual examination of the distribution of ICU stays for the study patients.

The equation for predicting ICU stay remaining after the threshold day was developed and internally validated using admissions from 2002 through 2005; 50% of these admissions were randomly allocated to be in the development data set and the remaining 50% were used in the internal validation data set. Because predictive models tend to be over specific for the database used for their development [35,36], we externally validated the model's accuracy using data for ICU admissions during 2006-2007. Inclusion and exclusion criteria and methods for assessing predictive accuracy were identical to those used for internal validation.

To determine which factors had the greatest impact on the model's predictive power, we separated total model variation into its components. This was accomplished by obtaining the sequential sum of squares due to each variable and then calculating their respective percentage of the total model's sums of squares.

### Assessment of Model Accuracy

We used several methods to assess the accuracy of aggregate predictions of ICU stay remaining after the threshold day [37]. First, we assessed the degree of correspondence between mean observed and mean predicted remaining ICU stay. A paired Student's t-test was used to assess the null hypothesis that the mean residual between observed and predicted remaining ICU stay was zero. Second, we calculated a coefficient of determination (r^2^) to measure the percentage of overall variability captured by the model for individual patients and across ICUs, respectively. Given the skewness of the data, we also examined the correlation between mean observed and mean predicted values using Spearman's rho. Third, we examined calibration by graphically displaying mean observed and mean predicted remaining ICU stay across 5% quantiles of observed values. Finally, we examined the model's accuracy when stratified by vital status at ICU discharge.

To test the utility of the model for prolonged ICU stay we compared the difference between mean observed and mean predicted total ICU stay based on the APACHE IV ICU day 1 model [10] and the predicted remaining ICU stay added to the threshold number of days (threshold day + predicted remaining ICU stay).

## Results

Data were collected in 138 ICUs at 54 hospitals from January 1, 2002 through December 31, 2007. When developing the predictive model we excluded 39 ICUs that did not collect daily physiological information, leaving data from 83 ICUs and 31 hospitals for analysis. Table 2 gives the characteristics of these hospitals and ICUs. For the 2002-2005 data set, 29 ICUs met inclusion criteria but did not collect data in 2006-2007. For the 2006-2007 data set, 6 ICUs met inclusion criteria but did not collect data in 2002-2005.

**Table 2:** Characteristics of hospitals and intensive care units that collected daily physiologic data

	#	%
# of Hospitals	31*	
Region		
NorthEast	3	9.7%
South	11	35.5%
Midwest	8	25.8%
West	9	29.0%
Bedsize		
≤ 200	8	25.8%
201 - 350	7	22.6%
351 - 500	6	19.4%
501 - 800	3	9.7%
> 800	7	22.6%
Teaching Status		
COTH^†^	6	19.4%
Non-COTH Teaching	11	35.5%
Non-Teaching	14	45.2%
# ICUs	83**	
Type		
Surgical	15	18.1%
Medical	13	15.7%
Mixed	29	34.9%
Coronary Care	11	13.3%
Cardiothoracic	7	8.4%
Neurologic	7	8.4%
Trauma	1	1.2%

* There were 30 hospitals in 2002 - 2005 and 21 in 2006 - 2007.

** There were 76 ICUs in 2002 - 2005 and 53 in 2006 - 2007.

^†^COTH = Council of Teaching Hospital

There were 380,953 ICU admissions from 2002 through 2007. Of these admissions, 13,311 (3.5%) were excluded because the patient was either admitted from or discharged to another ICU, and 2,201 (0.6%) because there was no discharge location information. A further 21,886 (5.7%) patients were excluded because they were admitted after coronary artery bypass surgery. This left 343,555 patients for determining a threshold value.

### Threshold for concern about a prolonged ICU stay

**Figure 1:**
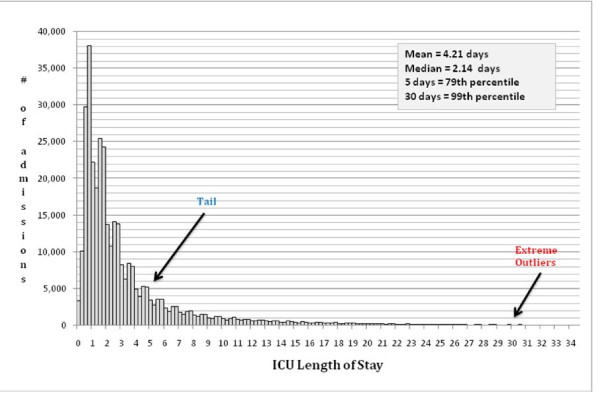
**Distribution of intensive care unit length of stay for 343,555 patients admitted from 2002 through 2007**.

The distribution of ICU length of stay for the 343,555 admissions that met inclusion criteria is shown in Figure 1. The mean ICU stay was 4.21 days and the median was 2.14 days, which is indicative of substantial skewness in length of stay. We identified ICU day 5 as the clinical threshold for concern about a prolonged ICU stay, as it allowed sufficient time to reflect complications or response to therapy and represented the 80^th ^percentile. An ICU stay > 30 days corresponded to the 99^th ^percentile, which delineates extreme outliers and confirms our choice for using it to truncate ICU stay.

**Figure 2:**
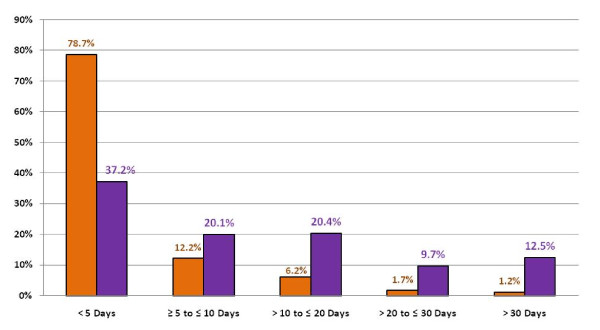
**Duration of intensive care unit (ICU) stay and its association with total ICU bed days**. Grey bars = % of ICU admissions. White bars = % of overall ICU days.

Figure 2 displays the percentage of ICU admissions and total ICU days respectively by ICU stay ranges. Admissions staying ≤ 5 days contributed 79% of all admissions but only 37% of all ICU days. Conversely, ICU stays > 30 days occurred 1% of the time, but resulted in 12.5% of all ICU days. Ranges of ICU stay between these two boundaries show that with an increasing ICU stay the percentages of total ICU days increase.

### Factors associated with an ICU stay < 5 days vs. ≥ 5 days

Patients with an ICU stay at or above the threshold of concern (≥ 5 days) were slightly less frequent in western U.S. and non-teaching hospitals; differences were small across ICU type. Table 3 compares the characteristics of admissions with an ICU stay <5 days compared to those with an ICU stay ≥ 5 days. Patients with an ICU stay at or above the threshold of concern (≥ 5 days) had a significantly higher day 1 mean Acute Physiology Score, APACHE IV predicted ICU stay on day 1, hospital stay before ICU admission, incidence of emergency surgery, ICU readmission, active treatment, and mechanical ventilation. Patients with a ≥ 5 day ICU stay were also more likely to have a chronic health condition, admission from the floor, other hospital, or a step down unit.

**Table 3:** Characteristics of patients with an intensive care unit (ICU) length of stay below (<5days) vs. above (> 5 days) a clinical threshold for concern about a prolonged ICU stay.

	Regular ICU Stays	Prolonged ICU Stays	P-value
# Admissions	270,487	73,068	
Age (years)	61.6 ± 17.7	61.9 ± 17.4	0.002
Acute Physiology Score - Day 1	36.2 ± 23.7	53.5 ± 25.7	<0.001
Day 1 ICU LOS Prediction	3.26 ± 2.04	5.80 ± 2.29	<0.001
Prior LOS (square root of days)	0.89 ± 1.27	1.26 ± 1.64	<0.001
Gender = Male	54.1%	55.1%	<0.001
Operative	31.7%	23.1%	<0.001
Emergency Surgery	5.2%	9.2%	<0.001
ICU Readmission	5.0%	10.6%	<0.001
Mechanical Ventilation on Day 1	27.9%	66.2%	<0.001
Received active treatment on Day 1	53.5%	82.5%	<0.001
≥ 1 Chronic health item	12.4%	16.4%	<0.001
Race			<0.001
White	65.9%	65.5%	
Black	13.1%	15.2%	
Latino	3.8%	3.8%	
Other	17.3%	15.5%	
Location Prior to ICU Admission			<0.001
Operating Room	9.9%	10.9%	
Recovery Room	21.8%	12.3%	
Emergency Room	38.0%	33.2%	
Floor	15.0%	22.9%	
Other Hospital	5.2%	8.3%	
Direct Admission	5.7%	4.2%	
SDU	4.4%	8.4%	
Visit			<0.001
1	95.1%	89.5%	
2	4.4%	8.8%	
≥ 3	0.6%	1.7%	
Top Five Diagnostic Groups*			
Congestive heart failure	3.7%	Bacterial pneumonia	5.2%
Gastrointestinal bleeding, Upper	3.1%	Congestive Heart Failure	3.6%
Rhythm disturbance	3.1%	Sepsis, pulmonary	3.1%
Drug Overdose	2.6%	Intracerebral hemorrhage	3.0%
Carotid endarterectomy	2.6%	Cardiac Arrest	2.9%

* Does not include 'miscellaneous" categories within a body system.

Table 3 also shows the five largest diagnostic categories for patients being discharged before ICU day 5 vs. patients remaining in the ICU on day 5 or longer. With the exception of congestive heart failure, the two groups had different diagnoses with the highest incidence being bacterial pneumonia, which accounted for 5.2% of all ICU stays ≥ 5 days. In general, diagnoses associated with an ICU stay ≥ 5 days were characterized by a tendency for less rapid improvement, complications, and prognostic uncertainty. A more detailed list of these diagnoses is shown in Table 4.

**Table 4:** Coefficients for the main effect variables* in the APACHE IV multivariable regression model that predicts remaining intensive care unit (ICU) stay after day 5.

Variable	Coefficient	p-value
**DAY 1**		
APS (per 10 units)	-0.37470	0.02
AIDS	0.76707	0.30
Cirrhosis	-0.69324	0.10
Hepatic Failure	-1.00013	0.02
Immunosuppression	-0.16578	0.41
Lymphoma	-0.79370	0.18
Leukemia or Myeloma	-1.22184	0.02
Metastatic Tumor	0.04277	0.89
Admitted from the Floor	0.02120	0.91
Admitted from the Operating/Recovery Room	-0.37637	0.63
Admitted from Other Hospital	-0.27897	0.27
Length of Stay before ICU Admission (per day)	-0.43308	0.22
Age (per 10 years)	-0.11960	0.41
(PaO_2 _: F_i_O_2 _)/10 on Day 1 (per 10 units)	-0.00285	0.99
Emergency Surgery?	-0.37896	0.26
Ventilated on Day 1?	-1.59366	<0.01
Unable to Assess Glasgow Coma Score on Day 1?	-2.43030	<0.01
Rescaled Glasgow Coma Score on Day 1	-0.09905	<0.01
Day 1 ICU Length of Stay Prediction (per day)	0.84742	<0.01
Readmission to ICU?	0.18283	0.44
**DIAGNOSTIC CATEGORIES (non-operative)**		
Airway obstruction	-0.36374	0.67
Aspiration pneumonia	-0.02422	0.96
Bacterial pneumonia	-0.60479	0.16
Cardiac arrest	-0.01874	0.97
Cardiogenic shock	0.91314	0.19
Congenstive heart failure	0.21795	0.63
Hyperglycemic hyperosmolar-nonketotic coma	-0.46909	0.52
COPD (emphysema/bronchitis)	-0.11881	0.81
Cardiovascular, other	0.72263	0.14
General, other	0.75895	0.19
GI bleeding, upper	-0.42147	0.34
GI, other	0.25619	0.64
Hepatic failure	-2.31722	<0.01
Hypovolemia/dehydration (not shock)	0.07455	0.92
Intracerebral hemorrhage	0.97105	0.04
Multiple trauma (excluding head trauma)	0.85690	0.10
Neurologic, other	0.46786	0.42
Drug overdose	-0.15812	0.80
Pancreatitis	0.06019	0.94
Pleural effusion	0.71845	0.37
Pulmonary edema (noncardiac, ARDS)	-1.15959	0.03
Pulmonary embolism	-0.35920	0.62
Renal, other	0.43602	0.49
Respiratory arrest	0.89615	0.07
Respiratory, other	0.47893	0.26
Rhythm disturbance	0.67577	0.26
Subarachnoid hemorrhage, intracranial aneurysm	1.46182	0.01
Subdural/epidural hematoma	-0.71143	0.31
Seizure (no structural disease)	-0.50347	.50
Sepsis, gastrointestinal	-0.25015	0.66
Sepsis, other location	-0.36255	0.53
Sepsis, pulmonary	-0.06425	0.90
Sepsis, unknown location	-0.49536	0.36
Sespsis, urinary tract	0.33721	0.56
Sepsis, cutaneous	-0.5287	0.51
Stroke	-0.59321	0.27
Head trauma with multiple other injuries	-0.55691	0.45
Head trauma only	1.79925	<0.01
**DIAGNOSTIC CATEGORIES (post-operative)**		
Aortic aneurysm, elective repair	1.06657	0.24
GI malignancy	2.07196	0.04
CABG with single valve surgery	4.09432	<0.01
Craniotomy or transsphenoidal procedure, neoplasm	1.56513	0.14
Cardiovascular surgery, other	3.95827	<0.01
GI obstruction	0.79922	0.42
GI surgery, other	2.59499	<0.01
GI, perforation	1.47170	.12
GI, vascular ischemia	0.96945	0.35
Multiple trauma including the head	-0.41853	0.73
Multiple trauma excluding the head	1.88865	0.05
Neurologic surgery, other	2.64227	<0.01
Peripheral ischemia (embolectomy, thrombectomy, dilation)	1.95534	0.07
Respiratory surgery, other	2.24947	0.01
Thoracotomy, malignancy	2.98145	<0.01
Aortic aneurysm, rupture	-0.22531	0.84
Subarachnoid hemorrhage (aneurysm, arteriovenous malformation)	2.16347	0.06
Subdural/epidural hemtoma	0.67618	0.54
Head trauma only	-0.00303	0.99
Valvular heart surgery	3.21080	<0.01
**DAY 5**		
APS taken on Day 5 (per 10 units)	0.75210	<0.01
Ventilated on Day 5?	2.22229	<0.01
Unable to Assess Glasgow Coma Score on Day 5?	3.94695	<0.01
(PaO_2 _: F_i_O_2 _)/10 on Day 5 (per 10 units)	-0.96110	<0.01
Rescaled Glasgow Coma Score on Day 5	0.19615	<0.01
Delta APS Day 4 - APS Day 5 (per 10 units)	0.11160	<0.01

No selection methodology was used.

*All variables except for the spline terms

**Figure 3:**
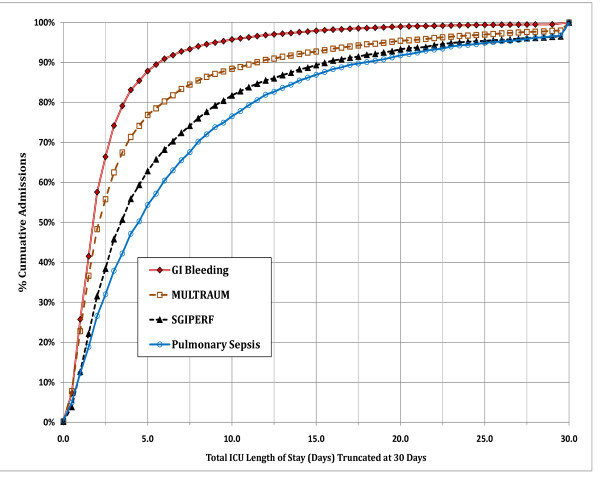
**Cumulative percentage of admissions across intensive care unit length of stay (truncated at 30 days)**. The diagnoses include Gastrointestinal Bleeding (GI Bleeding), Pulmonary Sepsis, Multiple trauma (MULTITRAUM), and surgery for Gastrointestinal Perforation (SGIPERF).

APS = Acute physiology score of APACHE III; AIDS = Acquired immunodeficiency syndrome; GI = Gastrointestinal; ARDS = Adult respiratory distress syndrome; CABG; Coronary artery bypass graft;

Figure 3 shows the cumulative incidence of ICU length of stay for four common diagnostic categories. For a large proportion of patients admitted for upper gastrointestinal (GI) bleeding ICU stay tends to be short. In contrast, the proportion of longer ICU stays is progressively larger and more skewed for patients admitted with multiple trauma, surgery for GI perforation and pulmonary sepsis, respectively. Table 5 shows the outcomes for patients being discharged before ICU day 5 vs. patients remaining in the ICU on day 5 or longer. The latter were more likely to have adverse outcomes and less likely to be discharged home.

**Table 5:** Outcomes for patients with an ICU stay below (< 5 days) vs. at or above threshold for concern (> 5 days) about a prolonged ICU stay.

	Regular ICU Stays	Prolonged ICU Stays	P-value
# Admissions	270,487	73,068	
Hospital Length of Stay: median & IQR	6.0 (3.3, 10.5)	18.6 (11.9, 30.7)	<0.001
Length of Mechanical Ventilation: median & IQR	1.0 (1.0, 2.0)	4.0 (1.0, 9.0)	<0.001
Hospital Mortality	10.7%	24.8%	<0.001
ICU Mortality	6.8%	14.7%	<0.001
% Discharged Home	68.4%	38.4%	<0.001

### Regression model to predict remaining ICU length of stay

There were 37,447 patients with an ICU stay ≥ 5 days that met inclusion criteria and were admitted to ICUs that collected daily data. They were divided as follows: 12,640 in the development data set, 12,904 in the internal validation data set, and 11,903 in the external validation data set. A multivariable linear regression model was developed to predict the outcome "length of stay remaining after ICU day 5". In aggregate the model included 99 variables: 90 of which were collected on ICU day 1, eight on ICU day 5, and one variable that reflected physiological changes from day 4 to day 5. The predictor variables and their multivariate relationship with this outcome are shown in Table 4.

**Figure 4:**
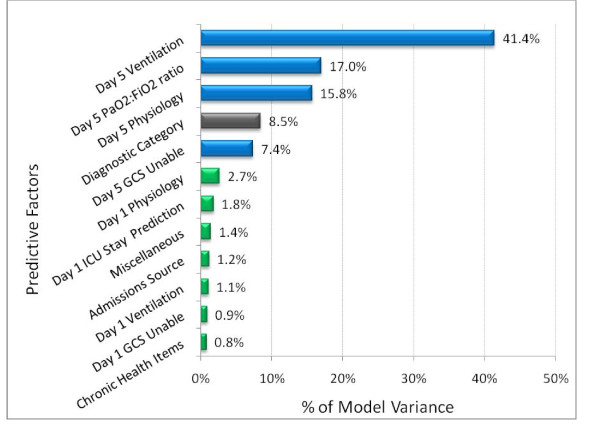
**Percentage of model variance attributable to factors used to predict intensive care unit length of stay remaining after day 5**. Physiology refers to the acute physiology score (APS) and rescaled Glasgow Coma Score. Rescaled PaO_2_/FiO_2 _ratio is defined in Appendix Table 1. GCS unable refers to inability to assess Glasgow Coma score due to sedation or paralysis. Diagnostic category includes 57 mutually exclusive diagnostic groups on day 5. Miscellaneous includes age, prior length of stay, emergency surgery, and ICU readmission.

For the 12,640 patients in the development data set mean predicted remaining ICU stay after day 5 was 6.87 days and mean observed ICU stay remaining after day 5 was also 6.87 days. The model had an r^2 ^= 20.2% across individuals and 44.3% across units. Spearman's rho was = 0.494 across individuals, analogous to an r^2 ^= 24.4%. The relative contribution of the variables used to predict ICU stay remaining after ICU day 5 is shown in Figure 4. ICU day 5 data accounted for 81.6% of the models overall explanatory power; day 5 mechanical ventilation for 41.4%, PaO_2_: FiO_2 _for 17%, other day 5 physiologic variables included in the APS score for 15.8%, and inability to assess Glasgow coma score due to sedation or paralysis for 7.4%. The remaining variables combined contributed 18.4% of the model's variation.

**Figure 5:**
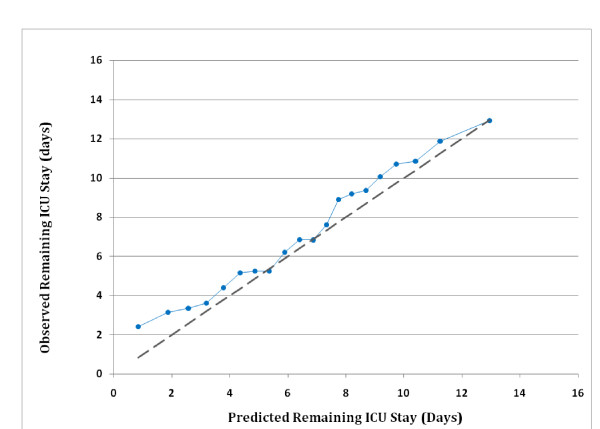
**Calibration curve comparing mean observed and mean predicted intensive care unit (ICU) length of stay remaining on ICU day 5**. The 11,903 patients in the 2006 to 2007 external validation set are divided into 20 equal-sized groups. The dotted line indicates perfect predictive ability (i.e. the observed means match the predicted means perfectly).

For the 12,904 patients in the internal validation data set mean observed remaining ICU stay was 6.87 days; mean predicted remaining ICU stay on day 5 was 6.85 days, a difference of < 1 hour. The mean residual value was not significantly different from zero (p > 0.05). For the 11,903 patients in the external validation data set, mean observed remaining ICU stay was 7.19 days and mean predicted remaining ICU stay on day 5 was 6.58 days, a difference of 14.6 hours (p < 0.001). When applied to the external data set the model had an r^2 ^= 18.2% across individuals and 43.3% across units; Spearman's rho was 0.486 across individuals (roughly equivalent to an r^2 ^= 23.6%). Figure 5 shows a calibration curve of observed length of stay over 5% quantiles of predicted values. Observed values deviate from predicted values mainly for the first two quantiles (lowest 10% of predicted values) and at two quantiles in the middle.

### Utility of the model for predicting lengthy ICU stays

**Figure 6:**
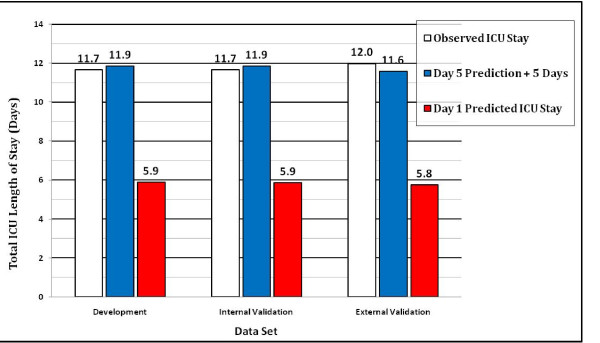
**Comparison of observed and predicted intensive care unit (ICU) length of stay**. Mean observed (ICU) length of stay (white bar), mean predicted length of stay based on the day 5 model [5 days + predicted remaining length of stay after day 5] (gray bar), and mean predicted length of stay based on day 1 model (black bar).

Comparison of mean observed and mean predicted total ICU stay using the APACHE IV ICU day 1 model versus the predicted remaining ICU stay added to the threshold number of days (5 days + predicted remaining ICU stay) demonstrates the usefulness of the model for predicting lengthy ICU stays. Figure 6 shows the mean ICU length of stay values for the development, internal validation, and external validation data sets, respectively. In each data set the sum of the day 5 prediction + 5 days was much closer to the observed ICU stay than the day 1 prediction. For the external validation data set, mean predicted total ICU stay was 11.58 days and mean observed ICU stay was 11.99 days, a difference of 9.7 hours (p < 0.001); using the day 1 prediction the difference between observed and predicted ICU stay was 149.3 hours (p < 0.001).

**Figure 7:**
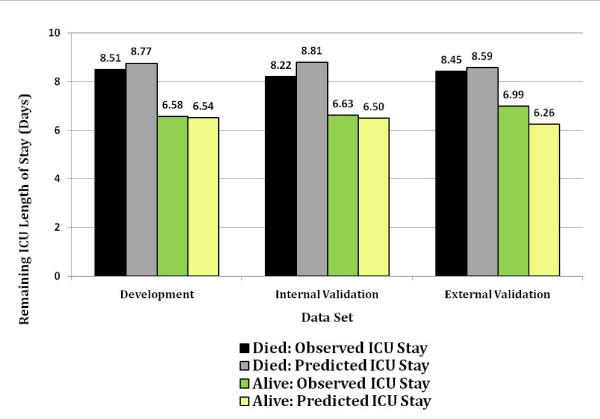
**Comparison of mean observed and mean predicted intensive care unit (ICU) length of stay remaining after day 5 among survivors and non-survivors at ICU discharge**. Black, died: Observed ICU stay. Gray, died: Predicted ICU stay. Green, alive: Observed ICU stay. Yellow, alive: Predicted ICU stay.

Figure 7 shows the mean observed and mean predicted remaining ICU stay stratified by ICU discharge vital status. There is close corroboration between observed and predicted mean ICU stay, regardless of whether or not the patient was discharged alive.

## Discussion

This study presents a model for predicting a prolonged length of stay for ICU patients. Because there is no uniform definition for a prolonged ICU stay, we identified a clinical threshold for concern about a prolonged ICU stay. We selected a ≥ 5 day threshold based on the study population's distribution of ICU length of stay and time needed to reflect a patient's early clinical course.

There were distinct differences between patients with an ICU stay < 5 days versus those with an ICU stay ≥ 5 days. Patients with an ICU stay ≥ 5 days had significantly higher severity of illness, frequency of mechanical ventilation, emergency surgery, and ICU readmission. Patients with an ICU stay ≥ 5 days accounted for 21% of all admissions but 63% of total ICU days; and their outcomes were uniformly poorer.

Based on the above findings, we developed and externally validated a multivariable regression model to predict ICU length of stay remaining after day 5. The variables used in the model were similar to those used in the APACHE IV day 1 model, but also included information captured during day 5. This additional information proved important because day 5 data accounted for 82% of the total predictive accuracy of the equation, leaving only 18% attributable to information collected on day 1.

We believe the model for predicting ICU stay remaining after day 5 accurately identified patients at risk for a prolonged ICU stay for several reasons: First, almost half (48.8%) of the model's explanatory power is accounted for by mechanical ventilation and inability to measure Glasgow Coma score due to sedation or paralysis on day 5. Prolonged (>96 hrs) mechanical ventilation [38,39] and sedation [40,41] have previously been associated with increased cost and ICU length of stay. Second, a prediction based in part upon a patient's physiology on day 5 reflects the impact of response to therapy, and or the development of complications, or both during the ICU stay [42,43]. Third, focusing on patients who remain in ICU for 5 days reduces predictive inaccuracy due to early deaths. Fourth, focusing on patients who remain in ICU for 5 days reduces inaccuracies due to differences in institutional discharge practices and length of stay variations caused by infrequent discharges between 10 pm and 7 am [44].

Previously published models of ICU length of stay developed for use in the U.S. [10,17,18,27,45], Western Europe [46], and Finland [34] all predict ICU stay on day 1 and have either not been tested or do not accurately predict a prolonged ICU stay. Inaccurate predictions of prolonged ICU stays using day 1 data alone has been attributed to prognostic uncertainty, complications, and variations in response to therapy.

Previous studies of patients with a prolonged ICU stay have also identified mechanical ventilation [6,45-47], higher severity of illness [5,45.46,47], and persistent physiological abnormalities (multiple organ dysfunction) [6] as risk factors. Infection has also been shown to pose a significant risk for a prolonged ICU stay [5,6,45,47]. Our analysis failed to identify infection because it was not included as a data element. Other previously identified variables that predict prolonged ICU stays including emergency surgery, trauma, prolonged pre-ICU hospital stay, and ICU readmission [5,6,45-47] were also used in our model.

Our study has several clinical implications. First, ICU clinicians who do not have access to advanced health information technology can use the most influential risk factors associated with a prolonged ICU stay to identify patients likely to have lengthy stays. Second, ICU clinicians with access to advanced electronic functionalities can use the model as a tool for improving ICU utilization. This is because patients who are likely to have a lengthy ICU stay can be referred for early mobility therapy [12,13], early discharge planning [48], palliative care consultation [11], or assessment for transfer to a long-term acute care facility [15,49]. Each of these interventions has been associated with a reduction in ICU stay. Third, the ability to compare case mix adjusted ICU stay can be used to compare the efficiency of resource use across ICUs. Because the APACHE IV ICU day 1 model accurately predicts length of stay across all patients [10], we recommend that it be used as a primary benchmarking tool. The model that predicts ICU stay remaining after day 5, however, provides additional information for benchmarking resource use for patients with prolonged ICU stays.

We do not recommend using this model to predict a prolonged ICU stay for individual patients. ICU day 5 prediction of a lengthy remaining ICU stay, however, can alert ICU clinicians to carefully consider patients within this group as candidates for interventions that might improve resource use.

The most important limitation of the model for predicting a prolonged ICU stay is its complexity. We believe this complexity reflects the large number of factors that determine a prolonged ICU stay. This complexity essentially mandates the use of automated data collection and calculation. Currently, the infrequent availability of advanced health information technology in most hospitals represents a major barrier to the model's widespread use [50]. As more institutions incorporate electronic medical records into their process flow, models such as the one described here can be of great value.

Our results have several additional limitations. First, the model's usefulness is probably limited to the U.S. because of international differences that impact ICU stay. These differences in ICU stay are also likely to adversely impact the use of ICU day 5 as a threshold for concern about a prolonged stay. Second, while capturing physiologic information on day 1 is too soon to account for the impact of complications and response to therapy, day 5 may still be too early to account for their effects. Previous studies indicate that more than half of the complications of ICU care occur after ICU day 5 [42,51]. Third, despite its complexity, the model fails to account for additional factors known to influence ICU stay. These include nosocomial infection [52,53], do not resuscitate orders [54], ICU physician staffing [54,55], ICU acquired paralysis [56], and ICU sedation practices [57]. Fourth, the model's greatest inaccuracy is the under-prediction of remaining ICU stays of 2 days or less (see Figure 5). We speculate that these findings might be explained by discharge delays aimed at avoiding night or weekend transfers [44] or the frequency of complications on ICU days 6 to 8 [51].

## Conclusions

A model that uses ICU day 1 and day 5 patient data was developed to predict ICU stay remaining after day 5. This model more accurately predicts prolonged ICU stays than a similar model that uses ICU day 1 data alone. The model can be used for performance benchmarking and as a tool for alerting clinicians to patients who may require early discharge planning.

## Competing interests

Cerner Corporation, Kansas City, MO, U.S.A. Cerner Corp, supported this study. Cerner Corp. owns the APACHE IV clinical database and markets a clinical information system that includes the APACHE system. AK is an employee of and has stock ownership in Cerner Corp. JZ provides consulting services and receives research support from Cerner Corp.

## Authors' contributions

AK and JZ conceived and designed the study. AK acquired the data and performed all statistical analyses. JZ drafted the manuscript. Both AK and JZ had full access to all of the data, made critical revisions, gave final approval to the manuscript, and take responsibility for the integrity of the data and the accuracy of the data analysis.

## Pre-publication history

The pre-publication history for this paper can be accessed here:

http://www.biomedcentral.com/1472-6947/10/27/prepub
